# Mediastinal Ectopic Pancreas Mimicking Lymphoma with Discordant Histology and Flow Cytometry: A Diagnostic Challenge

**DOI:** 10.3390/diagnostics16050797

**Published:** 2026-03-08

**Authors:** Guilin Ren, Hongfeng Wang, Haiqin Deng, Jianbin Chen, Li Wang, Qian Zhan, Jinxing Wu, Liwan Dai

**Affiliations:** 1 Department of Hematology, The First Affiliated Hospital of Chongqing Medical University, Chongqing 400016, China; 2 Department of Radiology, The First Affiliated Hospital of Chongqing Medical University, Chongqing 400016, China; 3 The Center for Clinical Molecular Medical Detection, The First Affiliated Hospital of Chongqing Medical University, Chongqing 400016, China; 4 Department of Respiration, The First Affiliated Hospital of Chongqing Medical University, Chongqing 400016, China

**Keywords:** ectopic pancreas, mediastinum, CT-guided percutaneous biopsy, flow cytometry, B-cell lymphoma, airway obstruction

## Abstract

**Background:** Mediastinal ectopic pancreas (EP) is an exceptionally rare entity that can mimic malignancy. Diagnosis is typically established post-operatively; pre-operative confirmation is challenging. **Case Presentation:** We describe a 28-year-old man presenting with life-threatening airway obstruction due to a progressive mediastinal mass, requiring emergency tracheal stenting. Diagnostic workup revealed a critical discordance: while CT-guided core biopsy confirmed benign ectopic pancreatic tissue, concurrent flow cytometry identified a monoclonal B-cell population with a high Ki-67 index (~86%), raising concern for a high-grade lymphoid process. However, no morphological evidence of lymphoma was found, and PET-CT showed only moderate metabolic activity (SUVmax 4.6), making an untreated aggressive lymphoma less consistent. The patient declined surgical resection. Management proceeded with a conservative strategy of structured clinical surveillance based on the benign histology. At 6-month follow-up, the patient remained clinically stable without chemotherapy, supporting the diagnosis of benign ectopic pancreas and suggesting the flow cytometric findings represented reactive “pseudo-monoclonality” secondary to inflammation. **Conclusions:** This case highlights mediastinal EP as a rare airway emergency and illustrates a major diagnostic pitfall: flow cytometric clonality and high proliferative fractions can occur in inflammatory settings and must not override benign architectural histology. When discordance persists and definitive tissue cannot be obtained, management should emphasize multidisciplinary review, deliberate specimen triage, and structured surveillance with predefined triggers for repeat higher-yield biopsy or surgical sampling and airway-stent reassessment.

## 1. Introduction

Ectopic pancreas (EP) is pancreatic tissue located outside the orthotopic pancreas and is a rare congenital anomaly, with a reported incidence of 0.5–13% in autopsy series [1,2]. The most common sites are the stomach (25–38%), duodenum (17–36%), and jejunum (15–21.7%) [3]. By contrast, mediastinal EP is exceedingly rare (<40 cases worldwide), usually arising in the anterior mediastinum as encapsulated cystic or solid-cystic lesions (Table S1) [4,5,6,7,8,9,10,11,12]. It appears to occur more often in younger patients, with a slight female predominance [13]. Clinical presentation is non-specific, ranging from chest pain and dyspnea to complete absence of symptoms [7]. Most published cases are single-patient case reports, with diagnoses established post-operatively on pathology [4,5]. Surgical resection remains the mainstay of treatment for symptomatic disease [5].

Mediastinal masses are frequently detected incidentally on chest imaging. Differential diagnosis is challenging, particularly distinguishing benign conditions (e.g., thymomas) from malignant neoplasms such as lymphoma, lung cancer, and mesothelioma [14,15]. Mediastinal lymphoma comprises approximately 10% of primary mediastinal tumors and 50% to 60% of all mediastinal malignancies [16]. Approximately 30–40% of patients are asymptomatic at early stages. As tumors enlarge, compression of the trachea, esophagus, or lungs may lead to dry cough, dysphagia, chest tightness, dyspnea, and, with superior vena cava involvement, superior vena cava syndrome [17,18]. Diagnosis of lymphoma relies on integrated morphology, immunohistochemistry, and flow cytometry interpreted by experienced hematopathologists, with molecular studies as needed. Incisional or excisional biopsy is preferred to obtain sufficient tissue; core-needle biopsy is an alternative when surgery is not feasible [19]. Endoscopic and imaging-guided approaches have improved access to mediastinal lesions, but obtaining sufficient diagnostic material can still be challenging in deep or anatomically complex masses. Accordingly, a comprehensive diagnostic strategy and timely multidisciplinary input are essential to enhance diagnostic yield and inform treatment planning [20,21].

Herein, we report a rare case of mediastinal ectopic pancreas causing life-threatening airway obstruction. This case is notable for a diagnostic discordance: flow cytometry raised concern for high-grade lymphoma, whereas core biopsy repeatedly showed benign ectopic pancreatic tissue. We discuss the potential pitfalls of such discordance, the mechanisms of reactive clonality in inflammatory lesions, and the management strategies for these complex mediastinal pathologies.

## 2. Case Presentation

**Figure 1:**
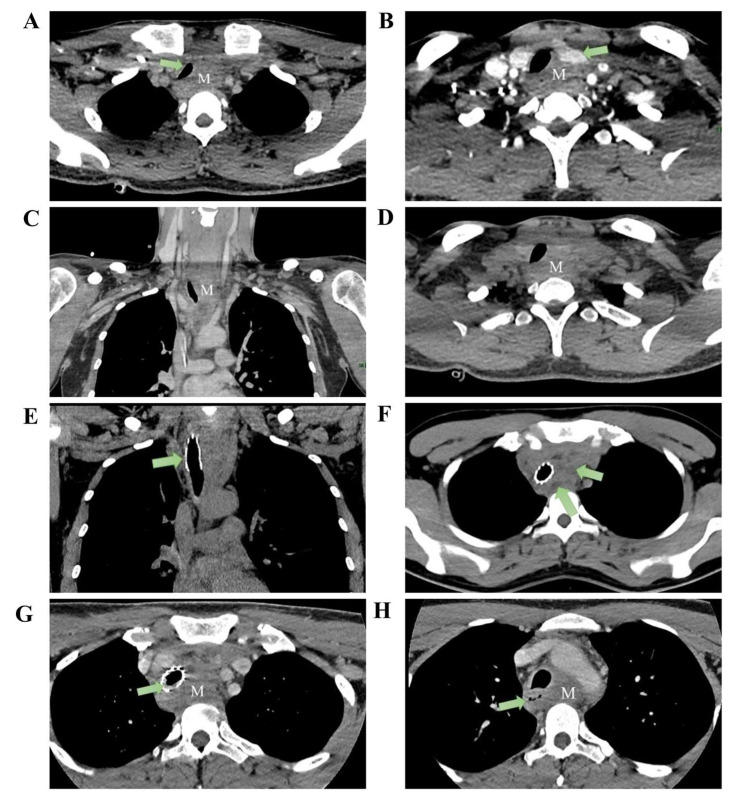
Pre-treatment imaging: CT reveals an anterior superior mediastinal mass (measuring approximately 3.9 × 2.3 cm) with the following features: (**A**) tracheal compression, (**B**) compression of the left thyroid lobe, (**C**) heterogeneous enhancement of the mass in the venous phase coronal section, and (**D**) axial plain CT scan shows no cystic changes within the lesion. Post tracheal stent placement: (**E**) CT coronal section demonstrates stent placement within the trachea. (**F**) Mass shows scattered cystic changes within its parenchyma. Two months post-treatment: (**G**) Mass demonstrates significant enlargement, measuring approximately 5.5 × 2.7 cm, with intraluminal granulation tissue hyperplasia observed within the tracheal stent. (**H**) Esophageal compression evident (M: mass).

A 28-year-old construction worker presented with a seven-month history of progressive dyspnea. He reported occupational dust exposure but denied smoking or alcohol consumption. His body mass index (BMI) was 22.5 kg/m^2^. Past medical history, family history, and drug allergies were unremarkable. Upon admission, physical examination revealed significant hypoxia and tachycardia: SpO_2_ 80%, respiratory rate 33 breaths/min, heart rate 115 bpm, blood pressure 122/73 mmHg, and temperature 36.2 °C. Lung auscultation revealed bilateral wheezing without focal neurological or cardiovascular deficits. Baseline laboratory evaluations, including blood cell counts, C-reactive protein (CRP), erythrocyte sedimentation rate (ESR), and renal and liver function tests, were within normal limits. Tumor markers, including CEA and CA19-9, were negative. Arterial blood gas analysis (performed on high-flow oxygen, FiO_2_ 58%) showed a PaO_2_ of 133 mmHg and PaCO_2_ of 45 mmHg. Spirometry revealed an FEV_1_/FVC ratio of 69.4%. Contrast-enhanced CT demonstrated circumferential soft-tissue thickening surrounding the proximal esophagus (3.9 × 2.3 cm; longitudinal extent: ~10 cm), causing esophageal narrowing and persistent tracheal compression. No mediastinal lymphadenopathy, pleural abnormalities, or chest-wall invasion were observed (Figure 1).

Given impending airway obstruction, 3-D CT of the tracheobronchial tree confirmed upper-tracheal stenosis with a ~3 cm narrowed segment. Emergency bronchoscopic balloon dilation and placement of a metallic stent were performed under general anesthesia using a supraglottic airway.

The balloon was inflated to 4.9 atm for 30 s (two cycles), followed by deployment of a partially covered metallic stent (40 mm × 16 mm) just below the vocal cords. The balloon was then inflated to 4.8 atm for 25 s (two cycles), and a second partially covered stent (40 mm × 16 mm) was deployed at the carina. Bronchoscopy demonstrated tumoral protrusion into the tracheal lumen with ~4 cm of stenosis. Forceps sampling was followed by two dilations and deployment of a 1.6 × 4.0 cm partially covered metallic stent (Figure 1E). Transbronchial needle aspiration, liquid-based cytology, and rapid on-site evaluation demonstrated only mild squamous epithelial atypia with scattered inflammatory cells and no malignant features. Dyspnea improved promptly after stenting. After symptomatic relief, upper gastrointestinal endoscopy identified a smooth-surfaced narrowing ~23 cm from the incisors, precluding adequate fine-needle aspiration. Considering the patient’s age, mass effect, and oncologic risk, the multidisciplinary team (MDT) recommended CT-guided percutaneous mediastinal biopsy. Histopathology demonstrated proliferative fibrosis with hyaline degeneration and chronic inflammatory infiltrates without malignancy. The patient declined further invasive procedures and was discharged.

**Figure 2:**
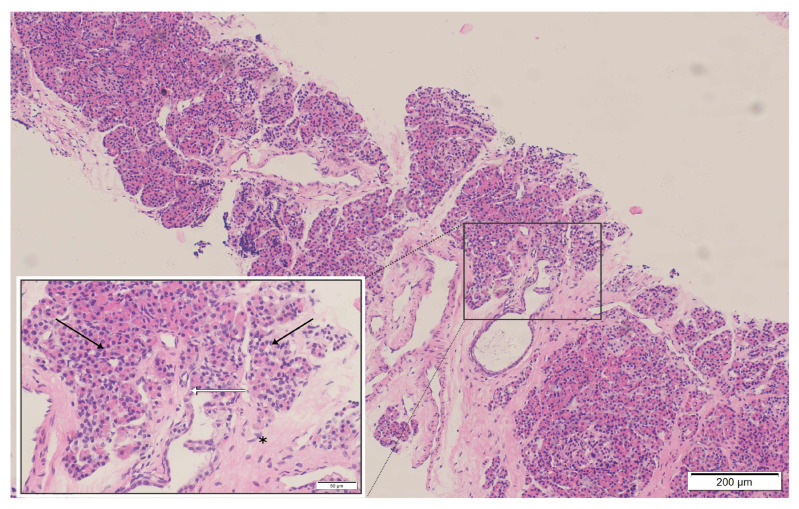
Histopathological examination of the mediastinal mass biopsy (H&E stain): The specimen reveals pancreatic acinar structures with a lobular arrangement (black arrows), composed of tightly packed basophilic acinar cells with benign morphology. Scattered pancreatic duct-like structures are present (white arrows). The epithelial components are embedded in dense fibrous connective tissue exhibiting hyaline degeneration (asterisk). These findings are consistent with benign ectopic pancreatic tissue; no evidence of malignancy or lymphoma infiltration is identified.

**Figure 3:**
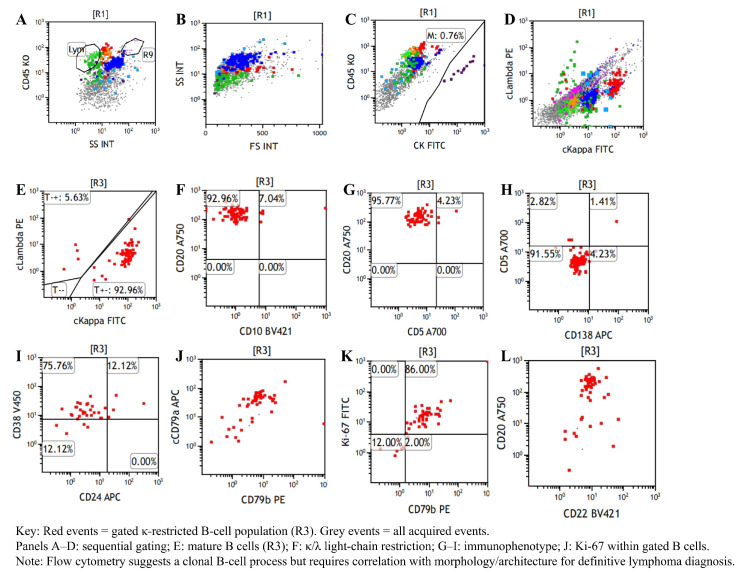
Flow cytometric immunophenotyping of the CT-guided mediastinal aspirate. The abnormal monoclonal mature B-lymphocyte population represents 0.87% of the total nucleated cells. (**A**–**C**): Identification of leukocytes via CD45/SSC and exclusion of CK+ epithelial cells. (**D**,**E**): Demonstration of significant kappa light-chain restriction (92.96% of the gated population), confirming a monoclonal nature. (**F**–**H**): Immunophenotypic characterization showing the abnormal B-cells express CD20^++^ and CD79b^+^, while lacking expression of CD10 and CD5. (**I**,**J**): Positive expression of CD38, CD24, and cCD79a, confirming a mature B-cell lineage. (**K**,**L**): Measurement of a Ki-67 index of 86.00% within the monoclonal gate.

Two months later, he re-presented with dysphagia and anterior-neck warmth and a self-limited low-grade fever (<38 °C) resolved with oral antibiotics. PET-CT showed SUVmax 4.6 at the mediastinal lesion with no uptake elsewhere (Supplementary Figure S1). CT confirmed a stable stent without restenosis, but the mass had enlarged to 5.5 × 2.7 cm with minimal/absent contrast enhancement (Figure 1G). PET-CT and bone-marrow assessment were unremarkable (Supplementary Figures S1 and S2), yet interval growth and location maintained concern for mediastinal lymphoma. A dual-guided strategy was employed, combining endoscopic ultrasound (EUS) with CT-guided percutaneous hollow-needle aspiration. EUS yielded limited material with few atypical cells. CT-guided sampling provided tissue in which histopathology established ectopic pancreatic tissue within fibrotic stroma with hyaline degeneration and chronic inflammation (Figure 2). Flow cytometry on the paired aspirate identified a small monoclonal mature B-cell population (CD20^bright^, CD22^+^, CD79a/b^+^, predominantly CD10^−^/CD5^−^) with κ-light-chain restriction (κ 92.96% vs. λ 5.63%; κ/λ ≈16.5) and a high Ki-67 proliferation index (~86% within the CD79b^+^ gate) (Figure 3), raising concern for a coexisting B-cell lymphoid process. However, no morphologic evidence of lymphoma was present on histology, precluding definitive subclassification. Metagenomic next-generation sequencing (mNGS) and routine laboratory tests did not identify infectious etiologies. Further staging and definitive treatment planning were constrained by the patient’s refusal of surgical excision or additional biopsy.

The patient was followed regularly in the outpatient clinic after discharge. At 6 months post-discharge, he remained clinically stable without recurrent dyspnea or new systemic symptoms. Importantly, he received no chemotherapy or corticosteroids during this period, and follow-up imaging showed no evidence of rapid progression. Ongoing surveillance was arranged, and tracheal stent management (including repeat bronchoscopic intervention) was planned if airway patency warranted. Because the patient continued to decline surgical excision, definitive histological resolution of the discordance remained unavailable; however, the clinical stability strongly favored a benign etiology.

## 3. Discussion and Literature Review

### 3.1. Ectopic Pancreas

Ectopic pancreas (EP) is pancreatic tissue located outside the orthotopic pancreas and is a rare congenital anomaly. The most frequently involved sites are the stomach (25–38%), duodenum (17–36%), and jejunum (15–21.7%) [1]. By contrast, mediastinal EP is exceptionally uncommon, possibly reflecting foregut developmental complexity. Since the first description by Shillitoe and Wilson in 1957, ~38 English-language cases have been reported (Table S1) [4,5,6,7,8,9,10,11,12]. Most publications comprise single-patient case reports, with diagnoses established post-operatively on pathology [5]. Reported mediastinal EP typically arises in the anterior mediastinum, has generally benign behavior with non-specific chest symptoms (or is asymptomatic), is often large (3.5–16.0 cm), carries a favorable prognosis after resection, and is predominantly cystic, leading to misdiagnosis as cystic teratoma [7,11,13,22,23]. Proposed mechanisms for the cystic morphology include obstructed exocrine drainage, inflammatory exudation, and hemorrhage [9]. The embryological origin remains debated. One view invokes aberrant ventral-foregut differentiation (precursor of the pancreas, trachea, and esophagus), whereas another proposes abnormal migration of pancreatic buds into the anterior mediastinum [5]. Given its rarity and non-specific presentation, pre-operative diagnosis is challenging. For symptomatic disease, surgical resection remains the mainstay of management. In contrast to most reports, our case secured a pre-operative diagnosis via CT-guided core-needle biopsy when endoscopic approaches were non-diagnostic, and airway-threatening presentation necessitated early bronchoscopic stabilization—points elaborated below.

### 3.2. Mediastinal Lymphoma and Diagnostic Challenges

Diagnosis in the mediastinum remains challenging owing to anatomical complexity and mass characteristics [16,18]. Although endobronchial ultrasound-guided transbronchial needle aspiration (EBUS-TBNA) is a valuable minimally invasive option, its diagnostic yield can be reduced by tumor fibrosis and low cellularity [24]. In our case, repeated EUS-FNA and EBUS-TBNA were non-diagnostic owing to inadequate sampling and limited patient tolerance [11]. This case demonstrates the limitations of endoscopic approaches for specific mediastinal tumors, especially those characterized by dense fibrosis or challenging anatomy. In such instances, small B-cell clones may be detected via flow cytometry even when they remain unsampled histologically [14,21]. Conversely, CT-guided percutaneous biopsy yielded sufficient tissue to confirm ectopic pancreas while identifying B-cell immunophenotypic abnormalities that were suspicious for, though not diagnostic of, non-Hodgkin lymphoma. These findings emphasize the importance of tailoring biopsy strategies to the anatomical and pathological complexity, and they highlight the necessity of multidisciplinary coordination and deliberate specimen triage in managing difficult mediastinal masses.

### 3.3. The Diagnostic Dilemma: Discordance Between Flow Cytometry and Histology

The discordance between flow cytometry raising concern for a clonal, highly proliferative B-cell population and histology demonstrating benign ectopic pancreas with chronic inflammation warrants consideration of multiple mechanisms within an integrated clinicopathologic framework [25]. First, sampling error remains a consideration in core-needle biopsies (CNBs). While CNB is highly specific, its sensitivity can be limited by tumor heterogeneity or fibrosis [26,27]. A small adjacent lymphoma focus (including a potential collision component or gray-zone-type lesion) could theoretically be missed by core biopsy yet sampled in the needle wash used for flow cytometry [26]. However, the absence of FDG-avid lymphadenopathy elsewhere and the patient’s stable clinical course over 6 months make a missed high-grade lymphoma less likely. Second, and most plausible in this context, is reactive clonality (pseudo-monoclonality). This phenomenon is a recognized pitfall in florid inflammatory conditions (such as the pancreatitis seen here), where flow cytometry may detect apparent light-chain restriction or prominent clonal B-cell populations despite benign histology [28,29,30]. Ectopic pancreas may undergo pancreatitis-like inflammatory changes, particularly when exocrine outflow is impaired, resulting in persistent local tissue injury and fibrosis [31,32]. Such chronic inflammation can provide sustained antigenic stimulation and promote reactive follicular/germinal-center hyperplasia [33]. Importantly, light-chain restriction is not invariably synonymous with lymphoma. Light-chain-restricted germinal centers have been documented in reactive lymphadenitis without overt B-cell neoplasia [34]. Accordingly, a monotypic/clonal B-cell signal on flow cytometry should be interpreted only in conjunction with tissue architecture and cytomorphology, rather than as standalone evidence of lymphoma [35,36]. Biologically, germinal centers are physiologically highly proliferative. Thus, high Ki-67 labeling is an expected feature of reactive hyperplasia, particularly when the analyzed gate is enriched for activated germinal-center B cells [37]. Consequently, an elevated Ki-67 fraction within a gated B-cell subset represents a potential mimic of malignancy rather than specific evidence of high-grade lymphoma [38,39]. Importantly, flow-cytometric Ki-67 represents the fraction of Ki-67-positive events within a selected gate and is not directly interchangeable with tissue-based Ki-67 via immunohistochemistry, which is interpreted in an architectural context. Technically, interpretation in inflamed, low-cellularity specimens is further complicated by artifacts. Factors such as low viability, hemodilution, and non-specific cytophilic antibody binding (e.g., Fc-receptor adsorption or dead-cell sticking) can distort κ/λ ratios [39,40]. These pre-analytical confounders underscore the necessity of strictly correlating flow cytometric “clonality” with architectural histopathology. Third, the presence of occult precursor lesions, such as tissue-based monoclonal B-cell lymphocytosis (t-MBL) or in situ follicular neoplasia (ISFN), cannot be entirely excluded. These entities are characterized by small clonal B-cell populations restricted to tissue without overt lymphoma masses [41,42,43]. Although such precursors could theoretically account for a clonal signal on flow cytometry, they are generally indolent findings and would be less likely to explain the markedly elevated Ki-67 fraction (~86%) observed within the gated B-cell population in this case. Therefore, the combination of apparent clonality and high proliferative fraction is more plausibly attributable to a florid inflammatory reaction with physiologically proliferative germinal-center B cells, together with the known interpretive pitfalls of flow cytometry in inflammatory/low-cellularity specimens [28,30,44].

### 3.4. Metabolic Characterization on PET-CT

PET-CT demonstrated only mild-to-moderate FDG uptake in the mediastinal lesion (SUVmax 4.6) [45]. Aggressive B-cell lymphomas often show intense hypermetabolism. For example, baseline diffuse large B-cell lymphoma has been reported with a median SUVmax of 24.35 (range 6.30–60.36) [46]. Crucially, there is a biological discordance in this case between the modest metabolic activity (SUVmax 4.6) and the extremely high proliferative index suggested by flow cytometry (Ki-67 ~86%). As discussed in Section 3.3, the Ki-67 value was derived from a gated B-cell subset and may reflect reactive biology and technical confounders in inflammatory/low-cellularity specimens. Therefore, PET-CT is interpreted only within this integrated framework. In true high-grade lymphomas with such high proliferation, glucose metabolism is typically markedly elevated. Although overlap exists, given that biopsy-proven lymphomas can occasionally present with lower SUV values [47], the observed activity falls squarely within the range described for inflammatory conditions, such as autoimmune pancreatitis (median SUVmax 5.0) [48]. Therefore, an SUVmax of 4.6 makes an untreated high-grade lymphoma less consistent, but it cannot exclude focal or indolent lymphoid processes, and must be interpreted together with histology, specimen quality, and clinical evolution [45,47]. In this case, the modest metabolic activity, benign architectural findings on core biopsy, and subsequent clinical stability collectively supported a conservative surveillance strategy rather than empirical lymphoma-directed therapy.

### 3.5. Clinical Implications and Management of Discordant Findings

Admittedly, the absence of surgical resection in this case leaves residual diagnostic uncertainty. Ideally, this discordance would have been resolved via surgical excision or repeat high-yield biopsy, as recommended by our multidisciplinary team to definitively exclude a focal lymphoid neoplasm (including gray-zone/collision scenarios). However, the patient declined further invasive interventions. Management was based on risk-benefit assessment. Core biopsy showed benign architecture, and PET-CT demonstrated only mild-to-moderate FDG uptake (SUVmax 4.6), supporting a conservative strategy. Nevertheless, the subsequent clinical course provides supportive, retrospective context for the benign interpretation. The patient has been closely monitored for 6 months without receiving chemotherapy or cytoreductive therapy. During follow-up, the mediastinal lesion remained radiographically stable. His respiratory status improved after airway stabilization and remained stable thereafter. While untreated aggressive B-cell lymphomas typically follow a rapidly progressive natural history, making prolonged stability less consistent with high-grade disease, this observation alone cannot fully exclude an indolent or focal lymphoid process that may be missed by limited sampling [35,41,49].

This case illustrates a practical management strategy for similar diagnostic challenges. When ancillary studies (like flow cytometry) conflict with reference-standard histopathology, we suggest a stepwise approach: (i) Reassessment: re-evaluate pre-analytical and analytical variables (e.g., viability, hemodilution, gating strategy) and correlate flow findings strictly with cytomorphology. (ii) Multidisciplinary review: coordinate radiology, thoracic/interventional teams, and hematopathology to optimize targeting and prospectively triage specimens (prioritizing cores for architecture and IHC ± molecular studies, and aspirates for flow cytometry), recognizing the limitations of small-volume sampling [35]. (iii) Risk stratification: if clinical/imaging concern persists (e.g., interval growth, rising metabolic activity, new lymphadenopathy, or systemic symptoms), escalate to higher-yield tissue acquisition (repeat core biopsy with increased cores and/or surgical biopsy/excision via mediastinoscopy/VATS when feasible; advanced bronchoscopic approaches may also improve tissue yield in selected patients) [50,51]. (iv) Structured surveillance: when definitive tissue cannot be obtained due to patient factors, implement short-interval follow-up with predefined triggers for re-biopsy or surgery. In our case, this approach avoided empirical lymphoma-directed therapy in the absence of diagnostic histology while maintaining patient safety through close monitoring.

## 4. Conclusions

In conclusion, we report a case of CT-guided biopsy-confirmed mediastinal ectopic pancreas with flow-cytometric suspicion of coexisting B-cell non-Hodgkin lymphoma, without definitive histopathological confirmation. This case highlights the diagnostic challenges posed by mediastinal masses, particularly when anatomical complexity or histological factors limit endoscopic or bronchoscopic sampling. It underscores the value of imaging-guided biopsy, prospective tissue triage (parallel sampling for morphology, immunohistochemistry, flow cytometry, and, when indicated, molecular testing), and timely multidisciplinary collaboration to optimize diagnostic yield in rare or ambiguous thoracic lesions. Any biological relationship between ectopic pancreas and lymphoma remains speculative and requires further investigation.

## Data Availability

The original contributions presented in the study are included in the article/Supplementary Materials; further inquiries can be directed to the corresponding authors.

## Supplementary Materials

The following supporting information can be downloaded at: https://www.mdpi.com/article/10.3390/diagnostics16050797/s1. Figure S1: PET-CT imaging of the patient: negative findings for lymphoma; Figure S2: Bone marrow and biopsy findings: negative histopathological results; Figure S3: Timeline of clinical symptoms and diagnostic findings of the patient; Table S1: Clinical characteristics of ectopic pancreas in the mediastinum: a case report overview.
