# Supplementary material for: Mediastinal Ectopic Pancreas Mimicking Lymphoma with Discordant Histology and Flow Cytometry: A Diagnostic Challenge
Source: Diagnostics (Basel). 2026 Mar 8;16(5):797. doi: 10.3390/diagnostics16050797 (PMC12985141; doi:10.3390/diagnostics16050797)
Supplement: Supplementary file 1 [file diagnostics-16-00797-s001.zip › diagnostics-4111692-supplementary.pdf]

Supplementary Material

1 Supplementary Figures and Tables

Supplementary Figure S1. PET-CT imaging of the patient: negative findings for lymphoma.

(A)

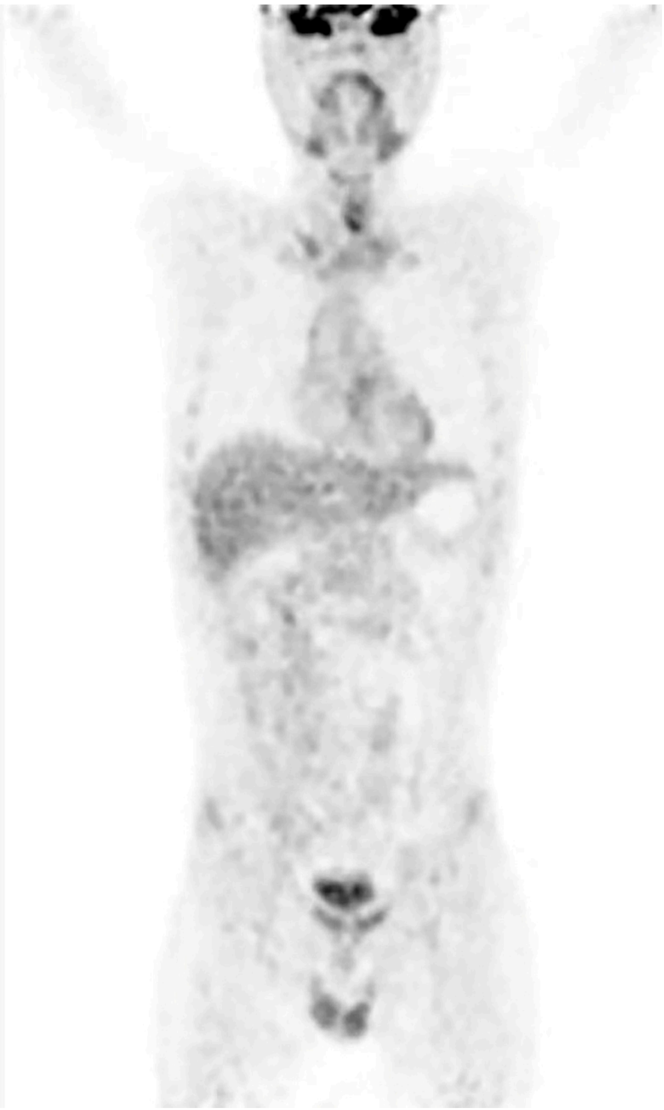

(B)

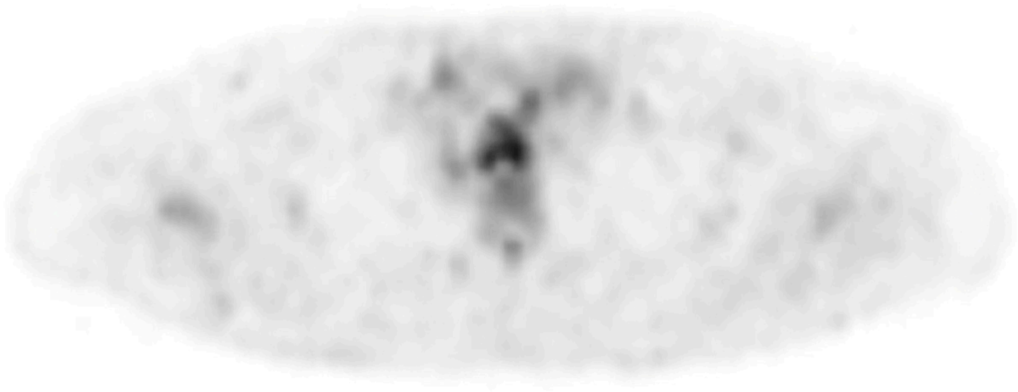

Supplementary Figure S2. Bone marrow and biopsy findings: negative histopathological results.

(A)

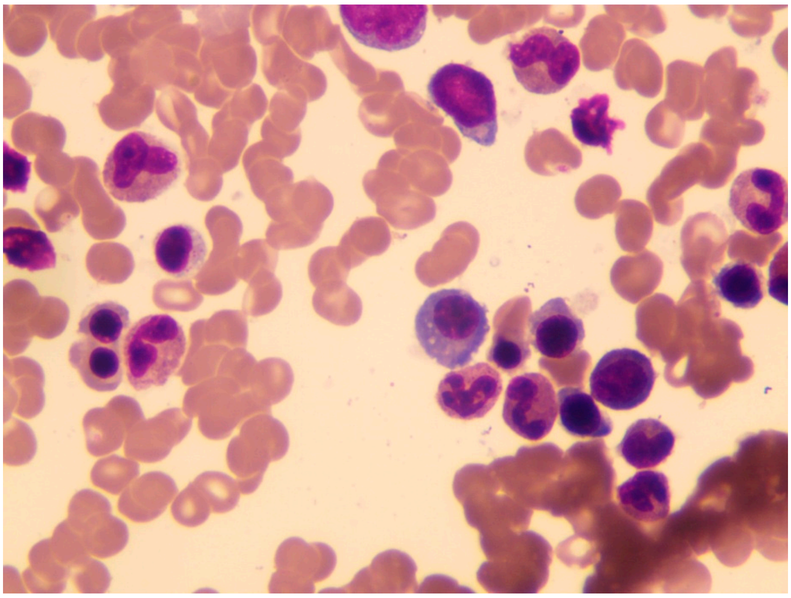

(B)

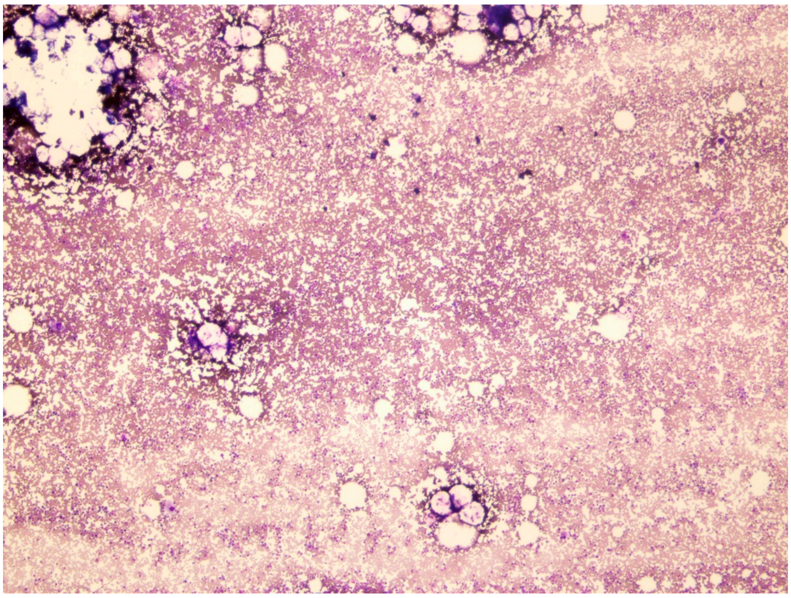

Supplementary Figure S3. Timeline of clinical symptoms and diagnostic findings of the patient.

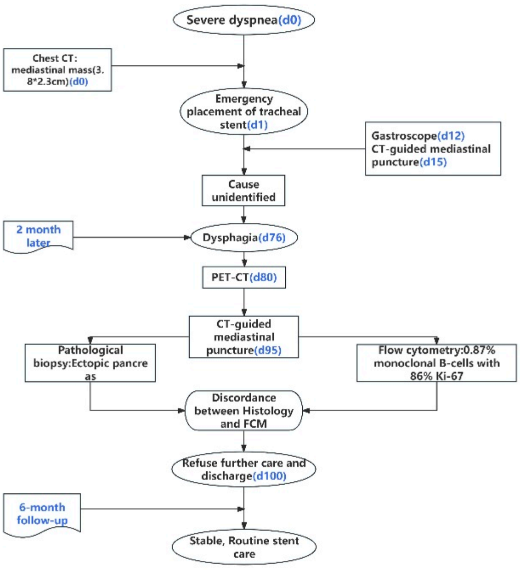

Tables

Table S1. Clinical characteristics of ectopic pancreas in the mediastinum: a case report overview.

SN	Reference	Gender	Age	Size (Cm)	Clinical presentation	Lymphoma Differential	Flow Cytometry	Pathology
1	Shillitoe et al. 1957	Female	15	5.5	Dyspnea, Night sweats	No	No	Benign
2	Carr et al. 1977	Female	57	10	None	No	No	Benign
3	Von Schweinitz et al. 1990	Male	5	5x5x5	Chronic Pneumonia	No	No	Benign
4	Perez-Ordonez et al. 1996	Female	16	12	None	No	No	Benign
5	Gong et al. 1997	Female	26	20x15	Chest pain, Cough	No	No	Benign
6	Gong et al. 1997	Female	26	4.3x1.3	Chest pain	No	No	Benign
7	Wu et al. 1998	Female	60	10x15	Chest pain	No	No	Benign
8	Cagirici et al. 2001	Female	45	10x8	Chest pain, Cough	No	No	Benign
9	Sentis et al. 2004	Male	44	10x8x7.5	Chest pain, Dyspnea	No	No	Benign
10	Yamato et al. 2005	Male	39	10x8	Chest pain	No	No	Benign
11	Al-Salam et al. 2006	Male	40	8x6x6	Cervical swelling	No	No	Benign
12	Wang et al. 2007	Male	17	12x12x4	Chest pain, Dyspnea	No	No	Benign

13	Wang et al. 2007	Female	24	10x8x4	Chest pain, Dyspnea	No	No	Benign
14	Ehricht et al. 2009	Male	25	15x15	Pneumonia	No	No	Benign
15	Chen et al. 2009	Female	32	13x16x8	None	No	No	Benign
16	Fayoumi et al. 2010	Male	51	10x7x5	Chest pain, Cough	No	No	Benign
17	Fayoumi et al. 2010	Male	42	10x5	Shoulder pain	No	No	Benign
18	Takemura et al. 2011	Female	21	3.5x3.5	Chest pain	No	No	Benign
19	Sandor et al. 2012	Male	32	4x4	Chest pain, Hemoptysis	No	No	Benign
20	Byun CS et al. 2012	Female	31	7x3x4	Chest pain, Cough, Hemoptysis	No	No	Benign
21	St Romain et al. 2012	Female	66	11x9	Chest pain	No	No	Malignant
22	Rokach et al. 2013	Female	22	5.1x3.8x2.3	None (Asymptomatic cervical mass)	Yes	No	Benign
23	Zhang et al. 2014	Male	15	7x4.5	Chest pain, Coughing, Fever	No	No	Benign
24	Zhang et al. 2014	Female	16	6	Throat discomfort, Neck swelling	No	No	Benign

25	Li et al. 2014	Male	18	16x12x9	Dyspnea, Stridor	No	No	Benign
26	Sibel et al. 2014	Male	23	6x8	None	No	No	Benign
27	Koh et al. 2015	Male	17	7.5x7x5.5	Cough, Sputum	Yes	No	Benign
28	Wu et al. 2015	Female	45	7.5x7x5.5	Hemoptysis	Yes	No	Benign
29	Mansi et al. 2017	Female	29 days	5x4x3.5	None	No	No	Benign
30	Snak et al. 2018	Female	21	6.7x7.5	Dyspnea, Chest pain	Yes	No	Benign
31	Njem et al. 2019	Female	2	20x16x3.5	Dyspnea	No	No	Benign
32	Zhao et al. 2019	Female	32	7x4x1.5	None	No	No	Benign
33	Sugata et al. 2021	Female	17	10	Chest pain	No	No	Benign
34	Iijima et al. 2021	Female	40	4.3x3.5x4.3	Precordial pain	No	No	Benign
35	Rochefort et al. 2020	Female	60	6 x 6	Hemoptysis	No	No	Benign
36	Sakurai1 et al. 2022	Male	18	2	None	No	No	Benign
37	Liu et al. 2023	Male	15	10.5x8.5x4	None	No	No	Benign

38	Berkarda Z et al. 2024	Female	22	1.8x2.0x2.2	Right shoulder pain, Dysphagia, Fever, Headaches	Yes	No	Benign
39	Index case	Male	28	5.2x4.1	Dyspnea	Yes	Yes	Benign
	Summary/Median	Female-23	26	1.8-20	Sympomatic-30	4	0	Benign-38
	Summary/Median	Male-16	24	2-15	Asymptomatic-9	2	1	Malignant-1
